# Phase II trial of radiotherapy after hyperbaric oxygenation with chemotherapy for high-grade gliomas

**DOI:** 10.1038/sj.bjc.6603342

**Published:** 2006-09-05

**Authors:** K Ogawa, Y Yoshii, O Inoue, T Toita, A Saito, Y Kakinohana, G Adachi, S Iraha, W Tamaki, K Sugimoto, A Hyodo, S Murayama

**Affiliations:** 1 Department of Radiology, University of the Ryukyus School of Medicine, 207 Uehara, Nishihara-cho, Okinawa 903-0215, Japan; 2 Department of Neurosurgery, University of the Ryukyus, Okinawa, Japan; 3 Department of Hyperbaric Medicine, University of the Ryukyus, Okinawa, Japan; 4 Department of Radiology, Naha City Hospital, Okinawa, Japan

**Keywords:** radiation therapy, glioma, glioblastoma, hyperbaric oxygen, chemotherapy

## Abstract

We conducted a phase II trial to evaluate the efficacy and toxicity of radiotherapy immediately after hyperbaric oxygenation (HBO) with chemotherapy in adults with high-grade gliomas. Patients with histologically confirmed high-grade gliomas were administered radiotherapy in daily 2 Gy fractions for 5 consecutive days per week up to a total dose of 60 Gy. Each fraction was administered immediately after HBO with the period of time from completion of decompression to irradiation being less than 15 min. Chemotherapy consisted of procarbazine, nimustine (ACNU) and vincristine and was administered during and after radiotherapy. A total of 41 patients (31 patients with glioblastoma and 10 patients with grade 3 gliomas) were enrolled. All 41 patients were able to complete a total radiotherapy dose of 60 Gy immediately after HBO with one course of concurrent chemotherapy. Of 30 assessable patients, 17 (57%) had an objective response including four CR and 13 PR. The median time to progression and the median survival time in glioblastoma patients were 12.3 months and 17.3 months, respectively. On univariate analysis, histologic grade (*P*=0.0001) and Karnofsky performance status (*P*=0.036) had a significant impact on survival, and on multivariate analysis, histologic grade alone was a significant prognostic factor for survival (*P*=0.001). Although grade 4 leukopenia and grade 4 thrombocytopenia occurred in 10 and 7% of all patients, respectively, these were transient with no patients developing neutropenic fever or intracranial haemorrhage. No serious nonhaematological or late toxicities were seen. These results indicated that radiotherapy delivered immediately after HBO with chemotherapy was safe with virtually no late toxicity in patients with high-grade gliomas. Further studies are required to strictly evaluate the effectiveness of radiotherapy after HBO for these tumours.

High-grade gliomas are relatively uncommon tumours, but cause a disproportionate number of cancer-related deaths because of their high lethality (Fine, 1994; Laperriere *et al*, 2002). Radiotherapy is a well established treatment for high-grade gliomas and plays an important role in the overall treatment of such patients, although it results in only a modest improvement in patient survival (Walker *et al*, 1978; Kristiansen *et al*, 1981). High-grade gliomas are generally considered to be radioresistant since a high fraction of tumour cells are hypoxic and resistant to radiotherapy as a result (Kayama *et al*, 1991; Rampling *et al*, 1994; Collingridge *et al*, 1999). Several reports have indicated that the median *p*O_2_ for high-grade gliomas studied under anaesthesia was approximately 5–7 mmHg with a significant proportion of observed *p*O_2_ values being <2.5 mmHg (Kayama *et al*, 1991; Collingridge *et al*, 1999).

Molecular oxygen has long been recognised to be a powerful modifier of cellular radiation sensitivity. The biological effect of ionising radiation has been reported to be increased approximately threefold when irradiation is performed under well-oxygenated conditions compared to anoxic conditions (Gray *et al*, 1953). Hyperbaric oxygenation (HBO) improves the oxygen supply to hypoxic tumour cells, and offers one approach to overcome tumour cell hypoxia such that this treatment has been used in combination with radiotherapy to treat malignant tumours (Jain, 1990; Hartmann *et al*, 2001). In this context, HBO is using oxygen as a drug by dissolving it in the plasma and delivering it to the tissues independent of haemoglobin. In some clinical trials and recent meta-analyses, significant improvements in both local tumour control and patient survival have been seen in patients with cancers of the head and neck and uterine cervix (Henk *et al*, 1977; Lindegaard *et al*, 1996; Overgaard and Horsman, 1996). Nevertheless, the delivery of simultaneous irradiation in combination with HBO is complex and time-consuming with some trials noting increased side effects (Jain, 1990; Dische, 1991; Dowling *et al*, 1992). As a result, the regimen of HBO in combination with simultaneous radiotherapy has not been used as a standard treatment for malignant tumours.

However, recent studies suggest that the oxygen tension within tumours improves during HBO with the increase being maintained for minutes following cessation of HBO (Kinoshita *et al*, 2000, Kunugita *et al*, 2001; Beppu *et al*, 2002). Many investigators have reported that malignant glioma cellular metabolism is anaerobic with the tumour exhibiting a lower oxygen consumption rate to normal white matter (Ito *et al*, 1982; Tyler *et al*, 1987; Mineura *et al*, 1994). The *p*O_2_ of normal brain tissue has been noted to decrease quickly after HBO treatment in animal experiments while the *p*O_2_ in high-grade gliomas falls more slowly after decompression as a result of the lower rate of oxygen consumption and the reduced blood flow to the tumour (Jamieson and van den Brenk, 1963). Therefore, in contrast to normal brain tissue, the *p*O_2_ within the tumour may remain elevated for a substantial period of time after decompression. The resultant increase in the oxygen tension within tumour cells would be predicted to sensitise high-grade gliomas to the cytotoxic effects of irradiation (Gray *et al*, 1953; Hall, 1994). This hypothesis suggests that radiotherapy immediately after HBO may increase the sensitivity of hypoxic tumour cells to radiotherapy without increasing the injury to normal brain tissue. Recently, several studies have shown the feasibility of this treatment regimen with HBO applied prior to radiotherapy for high-grade gliomas (Kohshi *et al*, 1999; Beppu *et al*, 2003; Ogawa *et al*, 2003). However, there has been little information regarding the clinical efficacy and safety of this treatment strategy.

In addition to radiotherapy, chemotherapy is often administered as a peri- or post-radiation treatment for patients with high-grade gliomas. Although the role of chemotherapy remains controversial, recent meta-analyses have demonstrated an increase in patient survival for patients treated with both chemotherapy and radiotherapy as opposed to those treated with radiotherapy alone (Fine *et al*, 1993; Stewart, 2002). Moreover, a recent randomised trial also demonstrated that the addition of concurrent chemotherapy (temozolomide) to radiotherapy resulted in a clinically meaningful and statistically significant survival benefit for patients with newly diagnosed glioblastoma (Stupp *et al*, 2005). Therefore, the standard of care has now shifted from radiotherapy alone to concurrent chemo/radiotherapy for these tumours.

Based on this background, we conducted a phase II study to evaluate the efficacy and toxicity of radiotherapy after HBO together with chemotherapy in adults with high-grade gliomas.

## MATERIALS AND METHODS

### Patient selection

This study was performed according to the guidelines approved by the institutional review board of our institution, with written informed consent being obtained from all patients. Patients aged 18 years of age or older with a histologically confirmed supratentorial glioblastoma, anaplastic astrocytoma, anaplastic oligoastrocytoma or anaplastic oligodendroglioma according to the World Health Organization (WHO) criteria (2000) who had not received prior treatment were eligible to take part in the study. All slides were reviewed by the same neuropathologist at our institution (SA). Other eligibility criteria included the following: Karnofsky performance status (KPS) score ⩾50%, normal bone marrow function (haemoglobin ⩾10 g dl^−1^, white blood cell (WBC) count ⩾3000 *μ*l^−1^, platelet count ⩾100 000 mm^−3^), normal renal function (serum creatinine <1.2 mg dl^−1^), normal liver function (AST <1.5 times normal, bilirubin <1.5 mg dl^−1^) and no evidence of cardiopulmonary disease or sinusitis.

### Hyperbaric oxygenation

Hyperbaric oxygenation treatment was performed in a multiplace hyperbaric chamber according to the following schedule: approximately 18 min of compression with air, 30–60 min of 100% oxygen inhalation using an oxygen mask at 2.8. Atmospheres absolute and approximately 18 min of decompression with oxygen inhalation. Following HBO, each patient was promptly moved to the treatment room and underwent radiotherapy. The period of time from completion of decompression to irradiation was consistently <15 min for each treatment fraction. All patients were gowned in regulation nonflammable pyjamas and no patients received sedation for each session.

### Radiotherapy

Radiotherapy began within 4 weeks of surgical resection. Each irradiation treatment was administered daily after HBO and was delivered with megavoltage machines of photon energies ⩾4 MeV. A head-holding device that was transparent to X-rays was used in order to ensure adequate immobilisation during radiotherapy and reproducibility. The treatment volume for both the initial volume and the boost volume was based on the preoperative computed tomography (CT) or magnetic resonance imaging (MRI) scans, and CT-guided treatment planning was required. The radiotherapy schedule consisted of a total dose of 60 Gy prescribed at the International Commission on Units and Measurements (ICRU) reference point and administered in 30 daily fractions over a period of 6 weeks (5 fractions per week). For the first 40 Gy, the treatment volume consisted of the contrast-enhancing lesions and surrounding oedema demonstrated on the CT or MRI scans with a 3 cm margin. After 40 Gy, the treatment volume was reduced and included the contrast-enhancing lesion (without oedema) apparent on the preoperative CT or MRI scans with a 2.5 cm margin. Anticonvulsive drugs, such as phenytoin or zonisamide, were administered orally to all patients during and after radiotherapy. Corticosteroids were used perioperatively, during the early phase of radiotherapy and as necessary thereafter. The corticosteroid dose had to remain stable for at least 1 week prior to entry into the study.

### Chemotherapy

Initial chemotherapy was administered concurrently with radiotherapy. One course of chemotherapy consisted of procarbazine 90 mg m^−2^ orally on days 1–14, nimustine (ACNU) 80 mg m^−2^ intravenously on day 1 and vincristine 0.5 mg m^−2^ intravenously on days 1 and 8. Cycles of chemotherapy were repeated at approximately 3 monthly intervals after radiotherapy up to a maximal total of four courses or until the tumour progressed or until the patient refused to receive further chemotherapy. All patients received antiemetics with granisetron and metoclopramide before ACNU administration.

### Patient evaluations

The extent of surgical resection was determined from the surgical records and postoperative CT or MRI scans by the neurosurgeons (YY and SA). Gross total resection represented a complete removal of the visible tumour, partial resection involved a 5–99% volume reduction and a biopsy sample indicated a <5% resection. All patients underwent a complete clinical history and physical examination, preoperative and postoperative CT and/or MRI scans, complete blood count (CBC), differential white cell count, platelet count, chemistry survey, neuropsychological testing and a chest radiograph before entry into the study. CBC, differential white cell count and platelet counts were obtained every week during treatment while a chemistry survey was performed every 2 weeks during treatment. Neurological examination and contrast-enhanced CT or MRI scans were obtained every 6–8 weeks during the first year. During the second year, patients in remission were evaluated with neurological examinations and contrast-enhanced CT or MRI scans at 3-month intervals. Neuropsychological assessment was conducted before radiation therapy and every 6 months thereafter or until the patient could no longer be tested.

### Assessment of response and toxicity

Assessment of response was based on postoperative CT or MRI scans that were obtained before and after radiotherapy and by neurological examination as described by Macdonald *et al* (1990). In brief, complete response (CR) was defined as the complete disappearance of all visible tumour, no steroid therapy and a neurologically stable or improved condition. Partial response (PR) was defined as a ⩾50% reduction in the product of the perpendicular diameters of the contrast-enhancing tumour in patients on a stable or decreasing dose of steroids who were neurologically stable or improved. Progressive disease (PD) was defined as a >25% increase in the product of the perpendicular diameters of the contrast-enhancing tumour or any new tumour seen on CT or MRI scans or neurologically worse, and the steroid dose being stable or increased. All other situations were defined as stable disease (SD). On the other hand, patients with no measurable contrast enhancing disease postoperatively were deemed to be non-assessable for response determination.

All toxicities were recorded and graded according to the common toxicity criteria of the National Cancer Institute, version 2.0. The dose of chemotherapy was decreased 33% for a nadir WBC count 1000–1500 *μ*l^−1^ or platelet count 20 000–50 000 *μ*l^−1^. For a nadir WBC count <1000 *μ*l^−1^ or platelet count <20 000 *μ*l^−1^, the dose of all agents was decreased by 50%. Chemotherapy was delayed if the WBC count was <3000 *μ*l^−1^ or the platelet count <100 000 *μ*l^−1^ at the beginning of each cycle until marrow recovery had occurred. Radiotherapy was delayed if the WBC count was <1000 *μ*l^−1^ or the platelet count <20 000 *μ*l^−1^ until marrow recovery had occurred. Chemotherapy was delayed if the AST level was elevated or if the direct bilirubin level was less than three times normal, until values fell to below twice the upper limit of normal. Chemotherapy was then restarted at 50% of the initial dose.

### Statistical analysis

Overall survival and progression-free survival rates were calculated actuarially according to the Kaplan–Meier method (Kaplan and Meier, 1958) and were measured from the day of surgical resection. Differences between groups were estimated using the log rank test (Mantel, 1966). Multivariate analysis was performed using the Cox regression model (Cox, 1972). A probability level of 0.05 was chosen for statistical significance. Statistical analysis was performed using the SPSS software package (version 11.0; SPSS Inc., Chicago, IL, USA).

## RESULTS

### Patient characteristics

Forty-one adults with newly diagnosed supratentorial high-grade gliomas were enrolled onto this trial between January 2000 and December 2003. The patient characteristics are detailed in Table 1. All patients were followed through December 2005 or until they died, and were evaluated for efficacy and toxicity of this treatment. The median follow-up time in the surviving patients was 57.1 months (range 28.1–70.6 months).

### Treatment delivered

All 41 patients completed a total dose of 60 Gy radiotherapy immediately after HBO with one course of concurrent chemotherapy. Thirty-three of 41 patients (80%) received further courses of chemotherapy after radiotherapy. The total courses of chemotherapy administered were four courses in 10 patients, three courses in six patients, two courses in 17 patients, and one course in eight patients.

### Response, survival and prognostic factors

Eleven patients had undergone a gross total resection and had no evaluable tumour at the initiation of protocol therapy. Therefore, 30 of 41 patients could be evaluated for response within 1 month after the completion of radiotherapy (glioblastoma: 22 patients, grade-3 gliomas: eight patients). Of these 30 patients, 17 (57%) had an objective response including four CR (13%) and 13 PR (43%). Twelve patients (40%) had SD and one patient experienced PD.

At the time of this analysis, 33 patients died (30 patients with glioblastoma and three patients with grade 3 tumours) and two patients with grade 3 tumours were alive with evidence of disease progression (follow-up, 36.9 and 28.1 months, respectively). The remaining six patients (one patient with glioblastoma and five patients with grade 3 tumours) had no evidence of tumour recurrence or disease progression (median follow-up, 68.2 months; range, 34.6–70.6 months). The median time to progression and the median survival time in glioblastoma patients were 12.3 months and 17.3 months, respectively. The 2-year actuarial progression-free survival and the 2-year actuarial overall survival rates in glioblastoma patients were 10 and 23%, respectively.

Table 2 indicates the results of univariate and multivariate analyses of potential prognostic factors on survival. On univariate analysis, histologic grade (*P*=0.0001) and Karnofsky performance status (*P*=0.036) had a significant impact on survival, and on multivariate analysis, histologic grade alone was a significant prognostic factor for survival (*P*=0.001).

### Toxicity

During HBO treatment, seven of 41 (17%) patients suffered middle ear barotrauma requiring tympanostomy with tube placement. Five of 41 (12%) patients had complaints of nausea requiring metoclopramide before each HBO treatment. No patients suffered convulsions during or after HBO.

The major acute toxic effects of chemotherapy (⩾grade 3) represent the most severe toxicity associated with the study treatment for each patient (Table 3). The percentage of grade 3–4 leukopenia, anaemia and thrombocytopenia were 44, 5 and 48%, respectively, with three patients (7%) requiring platelet transfusions and one patient (2%) requiring blood cell transfusions. Although grade 4 leukopenia and grade 4 thrombocytopenia occurred in 10 and 7% of all patients, respectively, these were transient with no patients developing a neutropenic fever or intracranial haemorrhage. Grade 3 nausea occurred in three patients (7%). Three patients (7%) developed grade 3 liver dysfunction and were treated conservatively. No other grade 3 or more severe nonhaematological toxicities were observed in the remaining patients. No severe late toxicities, such as intracranial haemorrhage and mental deterioration, were evident in the surviving patients at the time of analysis.

All patients exhibited alopecia within the treatment field. Regrowth of hair occurred over a time period of 4–8 months from the end of irradiation. Localised scalp erythema was seen in all patients although no patient experienced moist desquamation.

### Comparison of survival according to RPA criteria

We analysed our patients according to the recursive partitioning analysis (RPA) prognostic factors (Curren *et al*, 1993), which are based on a large Radiation Therapy Oncology Group (RTOG) database of patients with high-grade gliomas (Scott *et al*, 1998). Each RPA III, IV and V group in the current study had longer median survival time and higher 2-year survival than those of RTOG 90-06 results reported from Scott *et al* (Table 4).

## DISCUSSION

In recent years, radiotherapy immediately after HBO has been emerging as an attractive approach for overcoming hypoxia in cancer treatment (Al-Waili *et al*, 2005; Bennett *et al*, 2005; Mayer *et al*, 2005). Several experimental studies have indicated that the elevation in oxygen tension in the tumour is preserved for some time following HBO treatment, thereby allowing the administration of radiotherapy with an improved tumour/normal tissue oxygenation ratio (Kinoshita *et al*, 2000; Kunugita *et al*, 2001; Beppu *et al*, 2002). Using noninvasive MRI, Kinoshita *et al* (2000) demonstrated that the signal change related to the oxygen tension in SCCVII tumours decreased gradually but fell rapidly in muscle after HBO. Beppu *et al* (2002) conducted a direct stereotactic measurement of *p*O_2_ in glioblastoma tissue of patients under various conditions. The *p*O_2_ levels were significantly increased in both peritumoral and intratumoral tissues after HBO with a high *p*O_2_ level being maintained until 15 min after HBO in both regions. Moreover, performing radiotherapy immediately after HBO had a significant effect in experimental studies. Kunugita *et al* (2001) examined the effect of radiotherapy after HBO in SCCVII tumours (radiobiological hypoxic fraction: approximately 10%) subcutaneously transplanted into C3H/He mice using a growth delay assay. They noted a significant SCCVII tumour growth delay in the treated animals within 30 min after HBO with the tumour growth delay time being 1.61 times longer than that following radiotherapy alone. Moreover, oxygenation using HBO also enhanced the sensitivity to chemotherapy, because the hypoxic conditions prevalent in the tissue compromised the chemotherapeutic potential of almost all agents (Rampling *et al*, 1994; Al-Waili *et al*, 2005).

The current study suggested that radiotherapy administered immediately after HBO with chemotherapy may be promising for high-grade gliomas. Moreover, the administration of radiotherapy after HBO enabled the application of conventional fractionation of radiotherapy and the combination of modern radiotherapy techniques including patient fixation and three-dimensional conformal therapy. Several reports have also indicated the antitumour activity of irradiation immediately after HBO in conventionally fractionated irradiation (Kohshi *et al*, 1999; Beppu *et al*, 2003). Kohshi *et al* (1999) applied radiotherapy after HBO to high-grade glioma patients with residual disease. A total dose of 50–71 Gy in 20–30 fractions was administered, and 11 of 15 patients (73%) treated with radiotherapy immediately after HBO exhibited >50% tumour reduction. The median survivals in patients with and without HBO were 24 and 12 months, respectively, and were significantly different (*P*<0.05). Beppu *et al* (2003) applied radiotherapy after HBO with ACNU and interferon-beta for high-grade gliomas and 50% of glioblastoma patients exhibited >50% tumour reduction. The current study also indicated antitumour activity of conventionally fractionated radiotherapy delivered immediately after HBO with a total dose of 60 Gy in 30 fractions. In the 30 patients in whom it was possible to evaluate the therapeutic response (glioblastoma: 22 patients, grade 3 gliomas: eight patients), 17 (57%) had an objective response including four CR (17%) and 13 PR (43%). The median survival time has been reported to be 11–15 months in several phase studies for glioblastoma (Lassen *et al*, 1999; Choi *et al*, 2002; Wick *et al*, 2002; Grossman *et al*, 2003), and was 17.3 months in the current study. When using RPA analysis criteria, we found that the median survival times and 2-year survival rates of RPA III, IV and V classes in the current study compared favourably with patients in the RTOG databases who were treated in a randomised trial in the early 1990s (Table 4).

However, these favourable results demonstrated in the current study should be interpreted with caution. First, the patient characteristics were generally favourable with 25% of patients undergoing gross total resection, and 25% of patients having grade 3 gliomas. The median age of 57 was relatively young and the median KPS of 80 indicated a group with good performance status. Therefore, one might expect the outcome in this group to be better than the average described in the literature for glioblastoma. Second, there is an important difference between the current study and other previous reports (mostly using adjuvant chemotherapy) as patients have received concurrent chemotherapy. Comparisons of the recent results with patients receiving concurrent temozolomide in the Stupp trial (Stupp *et al*, 2005) indicate that the 2-year survival rates for glioblastoma are much the same: 23% in the current study and 26.5% in the Stupp trial. Therefore, it is necessary to strictly evaluate the true effectiveness of radiotherapy after HBO for these tumours in further studies.

The current study also indicated that the administration of radiotherapy immediately after HBO was a safe and practical procedure. With the routine use of anticonvulsive drugs and tympanostomy with tube placement in case of middle ear barotrauma, all 41 patients were able to complete a total dose of 60 Gy radiotherapy with conventional fractionation delivered immediately after HBO. With regard to chemotherapy, all 41 patients could receive one course of concurrent chemotherapy, and 33 of 41 (80%) patients received further chemotherapies. Whereas the spectrum of the nonhaematological toxicity was unremarkable, we observed frequent haematological toxicity with grade 3 and 4 leukopenia and thrombocytopenia (48% of all patients). One of the reasons may be the concurrent administration of chemotherapy and radiotherapy in this protocol (Kleinberg *et al*, 1999). However, the incidence of grade 4 haematological toxicity was less common and was transient. No patients exhibited neutropenic fever or intracranial haemorrhage and no serious late toxicity was evident at the time of this analysis. Several authors have also used a multidrug chemotherapy regimen containing ACNU, and they also found that grade 3–4 haematological toxicity was frequent, but of short duration with very few treatment-related deaths (Choi *et al*, 2002; Weller *et al*, 2003). These results indicated that the administration of radiotherapy immediately after HBO with chemotherapy was safe and practical with virtually no late toxicity for high-grade gliomas.

Regarding prognostic factors, many studies have reported that age, KPS, tumour size, histologic grade and the extent of surgical resection significantly affect the overall survival for high-grade gliomas (Laperriere *et al*, 2002; Buckner, 2003). However, the current study found no significant differences in overall survival with respect to age, KPS and extent of surgical resection on multivariate analysis, and only histologic grade was a significant prognostic factor for survival. The lack of any significant differences on survival according to age, KPS and extent of surgical resection may be the result of the small number of patients in the current study. Another possible reason, however, may be the improved radiosensitivity by HBO to large residual tumours as well as small residual tumours. A close association may exist between KPS, tumour size, and the extent of surgical resection because the residual tumour size determined by the operation type sometimes affects KPS (Beppu *et al*, 2003). Improvement in the radiosensitivity of large residual tumours may contribute to an increased response rate and overall survival for patients with low KPS and/or large residual tumours. Beppu *et al* (2003) also found no significant differences in the response rates with regard to age, KPS, and extent of surgical resection for high-grade gliomas treated with radiotherapy immediately after HBO. They concluded that radiotherapy after HBO could be applied to patients with poor prognostic factors and resulted in tumour response identical to patients with good prognostic factors. Further studies are required to determine which high-grade glioma patients might effectively benefit from radiotherapy delivered immediately after HBO for high-grade glioma patients.

In conclusion, performing radiotherapy immediately after HBO with chemotherapy was safe with virtually no late toxicity for high-grade gliomas. This treatment strategy may be promising and merits further investigation. Further prospective randomised trials are also warranted to accurately determine whether the delivery of radiotherapy immediately after HBO could be beneficial for patients with high-grade gliomas.

## Figures and Tables

**Table 1:** Patient characteristics

**Characteristic**	**No. of patients**
Total number of patients	41
	
*Gender*	
Male	24
Female	17
	
*Age (years)*	
Median	57
Range	22–73
	
*KPS (%)*	
Median	80
Range	50–100
	
*Histologic grade*	
3	10
4	31
	
*Tumour location*	
Frontal	15
Other	26
	
*Tumour size*	
⩽4 cm	10
>4 cm	31
	
*Mental status*	
Normal	35
Abnormal	6
	
*Radiation from symptom*	
⩽3 months	22
>3 months	19
	
*Neurologic function*	
Work	21
Other	20
	
*Histology*	
GM	31
AA	4
AO	6
	
*Extent of resection*	
Gross total	11
Partial	24
Biopsy	6

AA=anaplastic astrocytoma; AO=anaplastic oligodendroglioma; GM=glioblastoma; KPS=arnofsky performance status.

**Table 2:** Univariate and multivariate analysis of various potential prognostic factors in patients with high-grade gliomas

	***P*-value**		
**Variable**	**Univariate**	**Multivariate**	**RR (95% CI)**
Histologic grade (grade 3 *vs* grade 4)	0.0001	0.001	0.133 (0.038–0.460)
KPS (<70 *vs* ⩾70%)	0.037	0.336	0.698 (0.336–1.451)
Neurologic function (vork *vs* other)	0.138	—	—
Tumour location (frontal *vs* others)	0.187	—	—
RT from symptom (⩽3 *vs* >3 M)	0.452	—	—
Age (<50 *vs* ⩾50 years)	0.503	—	—
Mental status (normal *vs* abnormal)	0.528	—	—
Gender (female *vs* male)	0.670	—	—
Extent of resection (GT, PT *vs* BT)	0.622	—	—
Tumour size (⩽4 *vs* >4 cm)	0.909	—	—

BT=biopsy; CI=confidence intervals; GT=gross total resection; KPS=Karnofsky performance status; M=months; PT=partial resection; RR=relative risk.

**Table 3:** Major toxicities (*n*=41)

	**Grade 3**		**Grade 4**	
	**No.**	**%**	**No.**	**%**
Leukocytes	14	34	4	10
Haemoglobin	2	5	0	0
Platelets	17	41	3	7
Nausea	3	7	0	0
Hepatic toxicity	3	7	0	0

**Table 4:** Comparison of survival according to recursive partitioning analysis criteria

	**Current study**			**RTOG 90-06^a^**		
	**No. of patients^b^**	**Median survival (months)**	**2-year survival (%)**	**No. of patients**	**Median survival (months)**	**2-year survival (%)**
*RPA class*						
III	15	23.2	47	105	17.5	30
95% CI		12.0–34.4	21–73		15.6–20.2	21–39
						
IV	8	20.4	38	240	11.5	17
95% CI		15.1–25.7	4–72		10.8–12.7	11–22
						
V	15	12.6	20	150	7.4	8
95% CI		9.9–18.5	0–40		6.2–9.1	3–12

CI=confidence intervals; RPA=recursive partitioning analysis.

a Data from Scott *et al*, 1998.

b One patient of RPA class I, one patient with class II and one patient RPA class VI were not reported.
